# Rapid systemic spread and minimal immune responses following SIVsab intrarectal transmission in African green monkeys

**DOI:** 10.1172/jci.insight.183751

**Published:** 2024-12-06

**Authors:** Kevin D. Raehtz, Cuiling Xu, Claire Deleage, Dongzhu Ma, Benjamin B. Policicchio, Egidio Brocca-Cofano, Daniele Piccolo, Kathryn Weaver, Brandon F. Keele, Jacob D. Estes, Cristian Apetrei, Ivona Pandrea

**Affiliations:** 1 Department of Pathology and; 2 Division of Infectious Diseases, Department of Medicine, University of Pittsburgh, Pittsburgh, Pennsylvania, USA.; 3 AIDS and Cancer Virus Program, Frederick National Laboratory of Cancer Research, Frederick, Maryland, USA.; 4 Department of Infectious Diseases and Microbiology, Graduate School of Public Health, University of Pittsburgh, Pittsburgh, Pennsylvania, USA.; 5 Università di Pavia, Udine, Italy.; 6 Vaccine and Gene Therapy Institute and Oregon National Primate Research Center, Oregon Health and Science University, Portland, Oregon, USA.

**Keywords:** AIDS/HIV, Virology, AIDS vaccine, Adaptive immunity, Innate immunity

## Abstract

African green monkeys (AGMs) are natural hosts of SIV whose infection does not progress to AIDS. Since early events of infection may be critical to pathogenesis in nonnatural hosts, we investigated early SIV infection in 29 adult male AGMs intrarectally inoculated with SIVsab92018 (SIVsab) and serially sacrificed throughout acute into early chronic infection to understand patterns of viral establishment, dissemination, and their effect on disease progression. Using this model, we showed that foci of virus replication could be detected at the site of inoculation and in the draining lymphatics as early as 1–3 days postinfection (dpi). Furthermore, testing with ultrasensitive assays showed rapid onset of viremia (2–4 dpi). After systemic spread, virus was detected in all tissues surveyed. Multiple transmitted/founder viruses were identified, confirming an optimal challenge dose, while demonstrating a moderate mucosal genetic bottleneck. Resident CD4^+^ T cells were the initial target cells; other immune cell populations were not significantly altered at the site of entry. Thus, intrarectal SIVsab infection is characterized by swift dissemination of the virus, a lack of major target cell recruitment, and no window of opportunity for interventions to prevent virus dissemination during the earliest stages of infection, similar to intrarectal transmission but different from vaginal transmission in macaques.

## Abstract 2

We show that viral expansion and dissemination following intrarectal transmission are rapid and simultaneous in a natural host of SIV, as in progressive hosts, thus not accounting for infection outcome.

## Introduction

African nonhuman primates (NHPs) are the natural hosts of SIVs, with over 40 different SIVs known to circulate among a variety of NHP species in the wild (1, 2). Unlike Asian macaques, which are not naturally infected by SIVs (3–6) and progress to AIDS after SIV infection, African NHPs rarely show signs of disease progression to AIDS (7), and they generally maintain the homeostasis of their immune cell populations and effective immune responses despite active viral replication (7–9). This lack of disease progression is not due to some inherent lack of virulence of the infecting SIV strains, since chronic, high levels of viral replication equal to or greater than the levels typically observed in persons living with HIV persist for the remaining lifespan of the African hosts (10–13). Instead, natural SIV hosts have evolved various adaptations (14–16) to better manage the detrimental effects of SIV infection, rather than direct control or clearance of the virus by exquisite host immune responses (2, 17–24).

Previous research has indicated that the mechanisms by which natural hosts might avoid disease progression occur very early on during SIV infection, probably within days following transmission (17, 24–29), at the mucosal sites of viral entry and the initial sites of viral replication. However, relatively little has been known about the earliest events of mucosal transmission in natural hosts, such as African green monkeys (AGMs). Most of our knowledge of the early events of SIV mucosal transmission was derived from studies of intravaginal transmission of SIVmac in rhesus macaques (RMs) (30–35). These studies show that the virus must first cross the epithelial barrier, which is a significant impediment to transmission (30, 34, 36–38). After crossing the epithelium, the virus enters the submucosa and infects a small number of target cells. This initial target cell population is composed of CD4^+^ T cells, which can be more numerous at mucosal sites than other resident immune cells types (39). However, the initial target cell density is generally still not high enough to sustain long-term viral replication (R_0_ < 1). Thus, more CD4^+^ T cells must be recruited to the initial foci of replication by the virus-induced innate and inflammatory immune responses (37). Local dissemination through lymphatic drainage leads to even greater viral expansion when the virus reaches the draining lymph nodes (LNs), where there is an extremely high concentration of densely packed cells that might be targeted by the virus. Lymphatic dissemination also allows the virus to move into the bloodstream through the thoracic duct and thereby enter systemic circulation (30, 31). Several studies of SIVmac intrarectal (IR) transmission in RMs indicate that the virus follows the same general pattern of dissemination after IR exposure, albeit with different timing (40, 41).

The macaque vaginal model of mucosal transmission also predicts that the combination of the epithelial barrier, the paucity of resident CD4^+^ T cells in many regions of the vaginal mucosa, and the antiviral immune responses imposes a severe “mucosal bottleneck” on the number of transmitted/founder (T/F) viral variants that can successfully establish infection (42). Indeed, among 80% of heterosexual transmissions of HIV, only a single T/F genotype was identified to establish systemic infection (43, 44). Similarly, only 1–2 SIV T/F variants are normally transmitted in either adult or juvenile AGMs (26). Likewise, only a single T/F virus was found to establish infection in multiple AGMs in the wild (45). This implies that the general features of mucosal transmission are similar in natural hosts (i.e., AGMs) and pathogenic hosts (i.e., humans and macaques).

Nevertheless, there are several important characteristics of natural hosts that set them apart from pathogenic hosts that can affect transmission. Most notably, AGMs maintain significantly lower levels of CCR5^+^CD4^+^ T cell targets in blood, LNs, and mucosal sites than seen in humans and RMs (22, 46). In wild AGMs, while this reduced mucosal target cell availability is not sufficient to prevent vaginal SIVsab92018 (SIVsab) transmission to adults, it appears to limit oral transmission to juveniles and infants; these groups have even lower target cell levels than adults and exceedingly rare instances of SIV transmission, even during frequent breastfeeding (26, 46, 47). The target cell availability at the mucosal sites determining transmission efficacy is further supported by the observation that pig-tailed macaques, which have higher levels of CCR5^+^CD4^+^ T cells at mucosal sites than AGMs, can be IR infected with a dose of SIVsab 1 log lower than those needed to infect the adult AGMs (26).

To better understand the barriers to SIV transmission and the immunological determinants of early SIVsab IR infection and disease progression, we performed a detailed characterization of the early events occurring at the site of virus entry following IR inoculation in Caribbean AGMs, employed as a model of natural SIV infection (48, 49). Our goal was to characterize both the virological and immunological events of the earliest stages of mucosal transmission in AGMs, particularly at the site of inoculation. To this end, we used quantitative PCR (qPCR) analysis of both plasma and tissue sites proximal and distal to the sites of entry to track viral dissemination throughout the body from the site of inoculation. We also performed a thorough dissection of the rectum and distal colon to identify: (a) the initial foci of viral replication; (b) the population of founder cells in the mucosa; (c) changes in immune activation, immune cell recruitment, or cell death at the mucosal site; and (d) the mucosal bottleneck of virus transmission, by enumerating the T/F viral variants. We report that, upon IR inoculation, despite the limited mucosal target cell availability, SIVsab became established and disseminated systemically almost immediately. Meanwhile, the immune response to viral infection remained minimal, both at the site of inoculation and at more distal sites. Taken together, these findings indicate that virus amplification and spread occurred virtually concomitantly upon IR SIVsab challenge of AGMs, with no feasible window of opportunity for interventions aimed at preventing systemic infection.

## Results

### Study design.

To thoroughly characterize the very earliest stages of viral replication and dissemination, 29 adult male AGMs were IR challenged with 1 × 10^7^ copies of SIVsab. The inoculum consisted of diluted plasma collected from an acutely infected AGM, which had been established to be effective in a preliminary study and was shown to contain T/F variants (26). Twenty-seven of the 29 SIV-challenged AGMs became infected. The remaining 2 AGMs (AGM137 and AGM16) were not infected and therefore were excluded from all analyses. Infected AGMs were euthanized at well-defined time points covering both acute and early chronic SIV infection. They were divided into 5 groups based on their predicted viral loads (VLs): (a) preinfection (baseline); (b) pre–ramp-up (1–3 days postinfection [dpi]); (c) ramp-up (4–6 dpi); (d) peak (9–12 dpi); and (e) set point (46–55 dpi). Four unchallenged AGMs were included as a control group (Supplemental Figure 1; supplemental material available online with this article; https://doi.org/10.1172/jci.insight.183751DS1).

At the time of each necropsy, numerous compartments were sampled from each AGM. The collected tissues were processed for qPCR, histology, and flow cytometry (blood, gut, and LNs only). As the site of inoculation, the entire rectum and distal colon were excised and dissected into small (1 × 1 cm^2^) segments for qPCR and histology. Using this overall strategy, we increased the likelihood of capturing a rare instance of early viral replication at the site of inoculation, while being able to monitor the presence of virus throughout the body.

### Rapid onset of viremia upon IR SIVsab challenge of AGMs.

**Figure 1:**
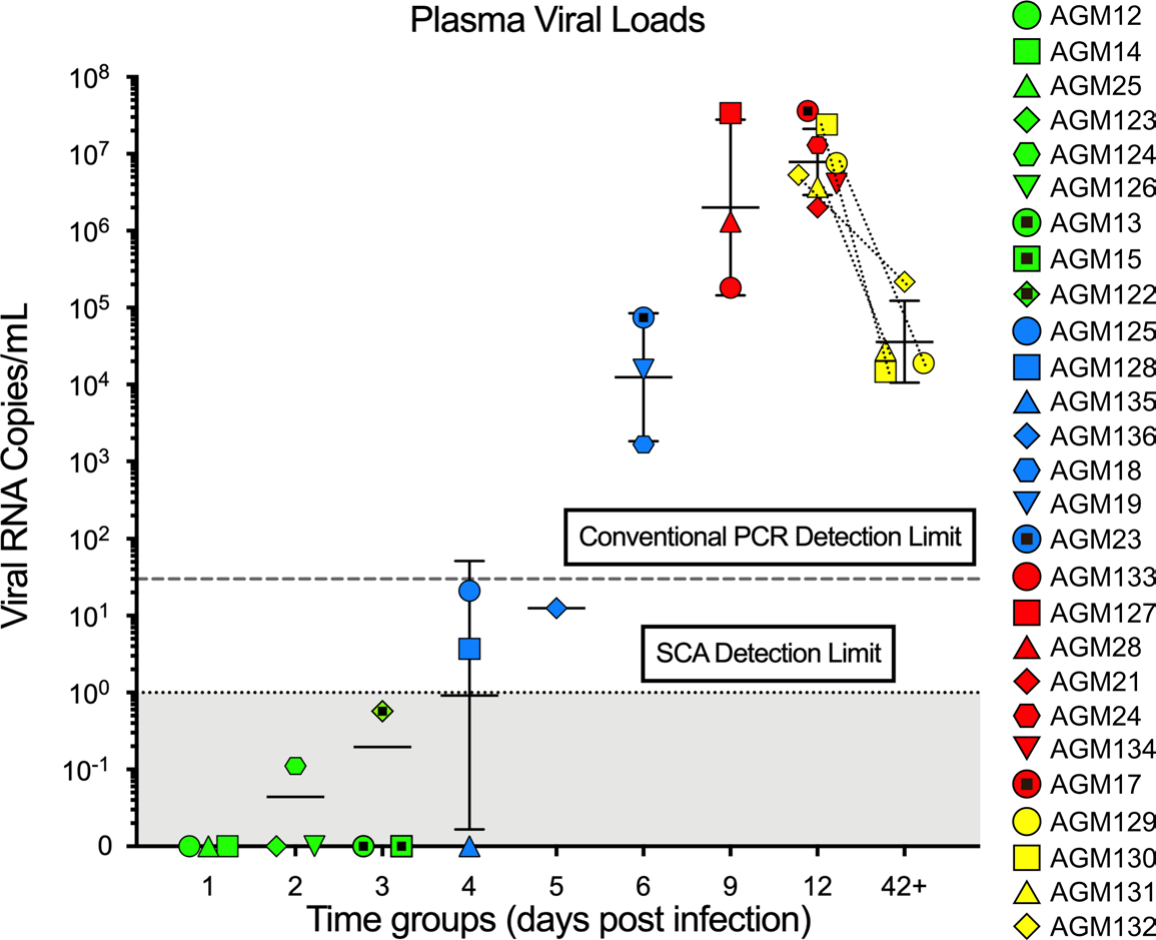
Plasma VLs in African green monkeys (AGMs) intrarectally (IR) infected with SIVsab. The plasma VLs are shown as log_10_ values. Each AGM that became infected after inoculation (*n* = 27) is represented by symbol with a unique color and shape combination as shown on the right. Blood samples from the chronically infected AGMs (yellow) were sampled both at peak infection and during early chronic infection, as indicated by the appearance of the symbols for those AGMs twice and the dotted lines connecting the same animal. Limits of detection for conventional and single-copy assays are shown by dotted horizontal lines, with the area below the SCA limit shaded in gray. The colors represent the different stages of infection, as defined by viral replication status: pre–ramp-up (green), ramp-up (blue), peak (red), and set point (yellow). The bars represent the geometric mean and SD of each group. AGMs that were negative for viremia are plotted directly on the *x* axis.

We monitored the early dynamics of viremia with a single copy assay (SCA), which has a theoretical limit of 1 viral RNA (vRNA) copy/mL (cp/mL) (50). At 4 dpi, 2 of 3 AGMs had VLs above 1 cp/mL (Figure 1). Beyond 5 dpi, all AGMs were viremic. In addition, 2 AGMs were viremic at 2 and 3 dpi, but virus was only detectable by testing very large volumes of plasma (AGM122 at 3 dpi had 5.8 × 10^–1^ vRNA cp/mL and AGM124 at 2 dpi had 1.1 × 10^–1^ vRNA cp/mL, respectively). The average plasma VLs then peaked between 9 and 12 dpi at 1 × 10^5^ to 1 × 10^8^ (geometric mean [GM], 20 × 10^6^) vRNA cp/mL. Finally, set point VLs were controlled to between 1 × 10^4^ and 1 × 10^5^ (GM, 3.6 × 10^4^) vRNA cp/mL. Note that the set point group AGMs were also sampled at 12 dpi and tested within the same range as the peak group AGMs.

### Cerebrospinal fluid (CSF) exhibits similar, but generally lower, VLs to plasma.

**Figure 2:**
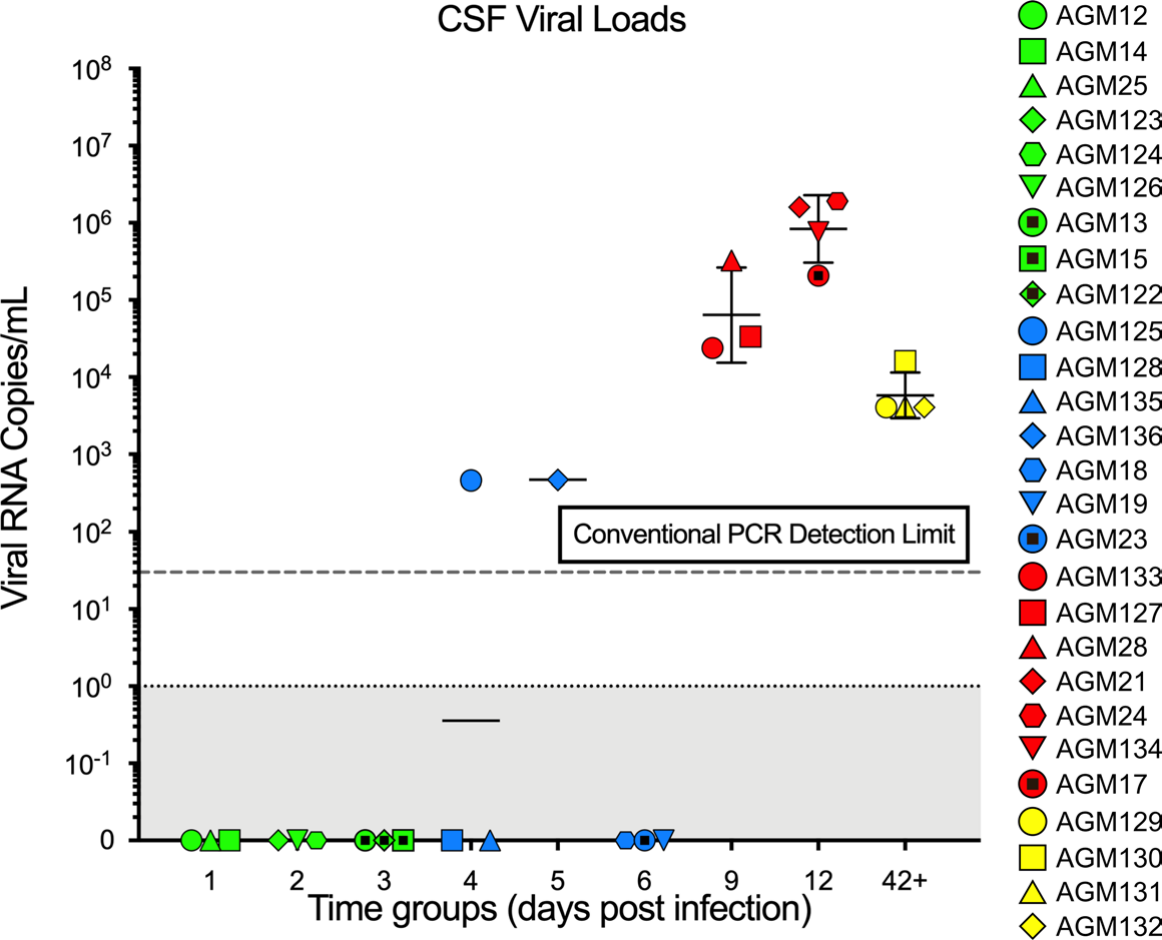
Cerebrospinal fluid (CSF) SIVsab viral loads in African green monkeys (AGMs) during acute and early chronic infection. The CSF viral loads are shown as log_10_ values. Each infected AGM (*n* = 27) is represented by a unique color and shape combination as shown on the right. The limit of detection for conventional qPCR (30 viral RNA copies/mL plasma) is indicated by a dotted line. The colors represent the different stages of infection as defined by viral replication status: pre–ramp-up (green), ramp-up (blue), peak (red), and set point (yellow). The bars represent the geometric mean and geometric SD of each group. AGMs that were negative for viremia are plotted directly on the *x* axis.

Due to limited volume*,* CSF was assessed by conventional qPCR but not SCA, which showed VLs with GMs of 6.0 × 10^4^ vRNA cp/mL at 9 dpi and 8.0 × 10^6^ vRNA cp/mL at 12 dpi, respectively. The VLs of the set point samples ranged from 1 × 10^3^ to 1 × 10^4^ (GM 8 × 10^3^) vRNA cp/mL. Two other CSF samples tested positive: AGM125 with 5 × 10^2^ vRNA cp/mL at 4 dpi and AGM136 with 5.0 × 10^2^ vRNA cp/mL at 5 dpi (Figure 2). Therefore, CSF testing supported the rapid systemic dissemination of the virus and further spread into the CSF, though it was unclear if the virus first entered the CSF through the blood or the lymphatics. Previous studies in macaques and more recent studies in humans have shown that, while VLs are typically lower in the CSF, the overall dynamics of viral burden in the CSF can mirror what was observed in the plasma in this study (51–54).

### Rapid systemic dissemination of SIVsab from the site of inoculation.

**Figure 3:**
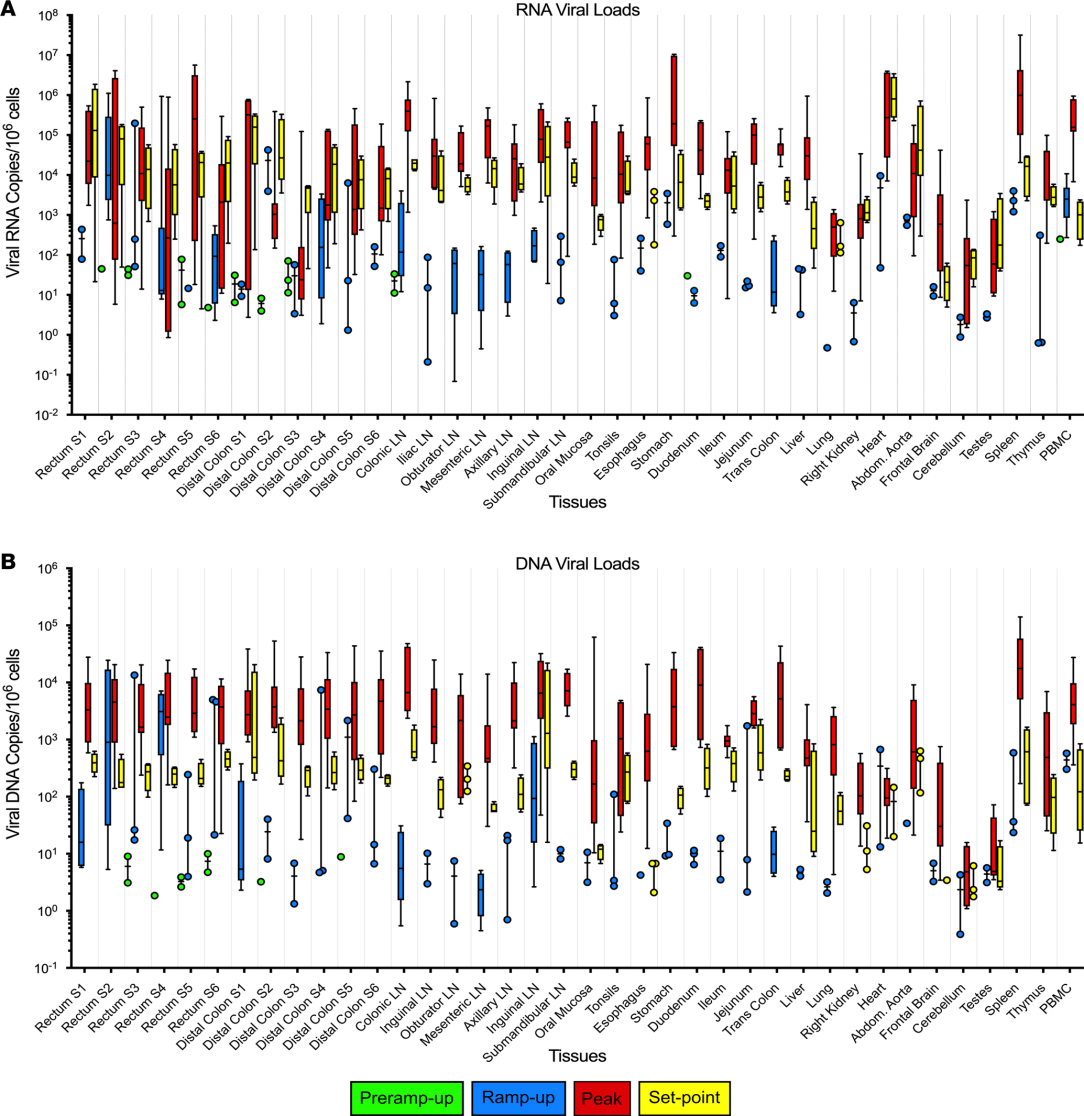
Total viral RNA and DNA in tissues of SIVsab-infected African green monkeys (AGMs). (**A** and **B**) The total viral RNA (**A**) and DNA (**B**) from each of the 38 tissues collected from the infected AGMs tested (*n* = 27) are shown, with dotted lines delineating each individual tissue. Viral loads are shown on a logarithmic scale and represent the total number of SIV genome copies per 1 × 10^6^ cells. The names of the tissues are listed below the *x* axis. The data are shown as box-and-whisker plots displaying the median, 1st and 3rd quartiles, and the minimum/maximum outliers. The colors represent the different stages of infection as defined by viral replication status: pre–ramp-up (green), ramp-up (blue), peak (red), and set point (yellow). For the rectum and distal colon, S1-S6 indicates from which strip of tissue the section was taken.

To examine the tissue replication kinetics, both within the site of inoculation and distally, vRNA (Figure 3A) and vDNA (Figure 3B) from multiple tissues were quantified via qPCR. In sections collected from AGMs in the pre–ramp-up group (1–3 dpi), the initial foci of replication yielded both detectable vRNA and vDNA, with the vRNA being more readily detectable (15 vRNA^+^ tissues versus 9 vDNA^+^ tissues). The pre–ramp-up group yielded multiple (AGM25, AGM13, AGM124, and AGM122) rectum or distal colon sections that contained either vRNA or vDNA, while AGM126 yielded only 1 vRNA^+^ section. Furthermore, in the pre–ramp-up AGMs, SIVsab vRNA was additionally detected in the draining colonic LNs (AGM15, AGM122), along with a sole instance of vRNA in the PBMCs (AGM123). We also detected vRNA in the colonic LNs of the ramp-up group (AGM125, AGM128) and the duodenum (AGM125), while vDNA was found solely at the site of inoculation. Taken as a whole, tissue VLs from the pre–ramp-up AGMs were low, with 1 × 10^0^ to 1 × 10^1^ (GM, 2.0 × 10^1^) vRNA copies/1 × 10^6^ cells (Figure 3A) and only 10^0^ (GM, 5.0 × 10^0^) vDNA copies/1 × 10^6^ cells (Figure 3B).

After the pre–ramp-up stage, while viremia was detected in the majority of the AGMs (4–6 dpi), of the 38 tissue types tested, 37 had detectable but highly variable levels of vRNA (1 × 10^–1^ to 1 × 10^5^ vRNA copies/1 × 10^6^ cells) and 36 had detectable levels of vDNA 1 × (10^–1^ to 1 × 10^4^ vDNA copies/1 × 10^6^ cells). Even immune-privileged sites like the testes and the brain tested positive for both vRNA and vDNA, although they had the lowest VLs on average. By the peak of infection, vRNA and vDNA were detectable in all tissues, at 1 to 1 × 10^7^ vRNA copies/ 1 × 10^6^ cells and 1 to 1 × 10^4^ vDNA copies/1 × 10^6^ cells, respectively. When viral replication reached its set point during the early chronic stage of infection, VLs in all tissues fell to 1 × 10^0^ to 1 × 10^6^ vRNA copies/1 × 10^6^ cells and 1 × 10^0^ to 1 × 10^4^ vDNA copies/1 × 10^6^ cells (Figure 3), respectively.

### Multiple T/F viruses established infection in each AGM, though a mucosal bottleneck still occurred.

**Figure 4:**
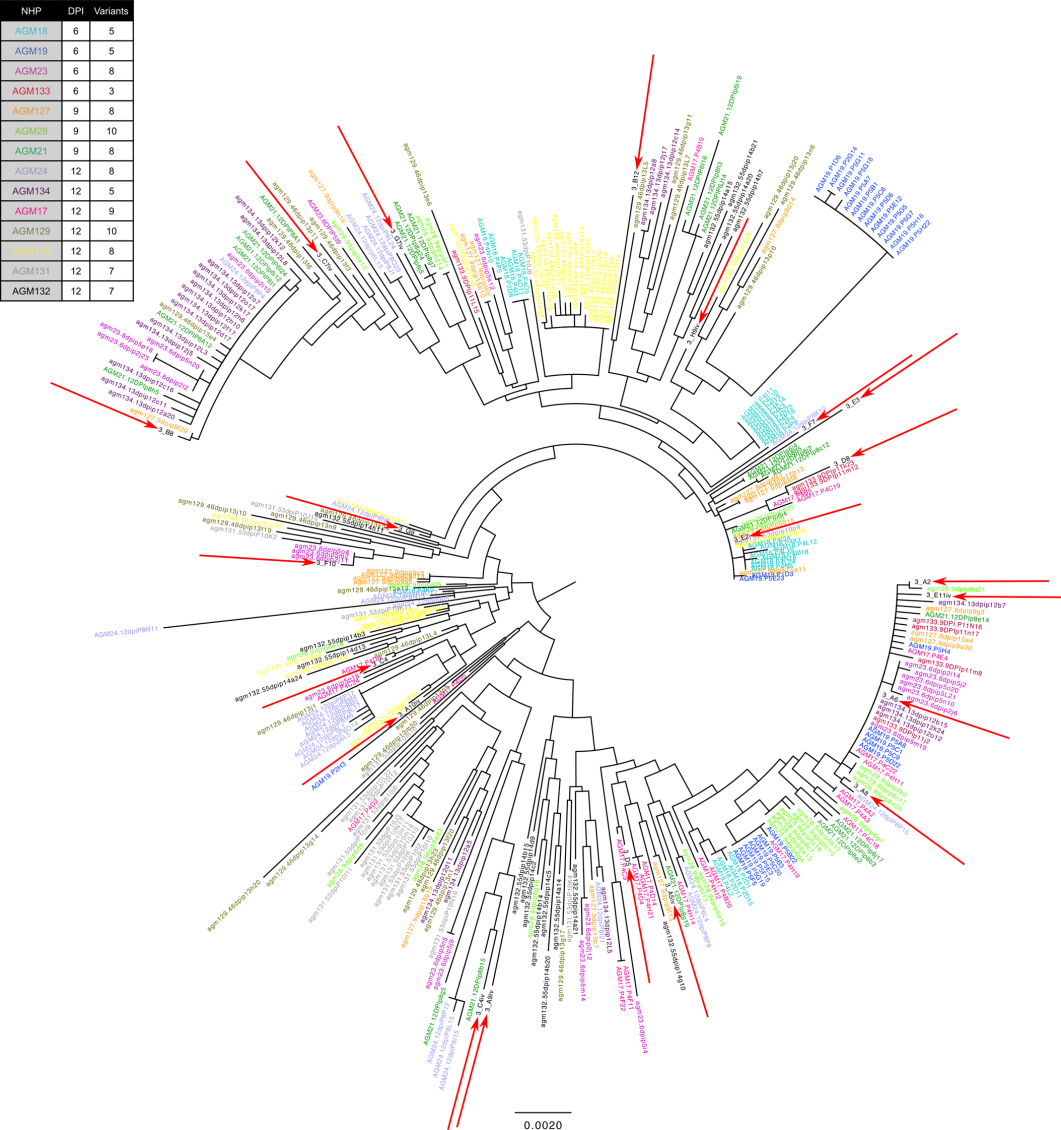
Single genome amplification of SIVsab transmitted/founder (T/F) viruses. The totality of T/F viruses from AGMs (*n* = 14) listed on the left are shown as a circular phylogenetic tree. The 14 AGMs shown here represent AGMs for which viremia was reliably confirmed by conventional qPCR. The color of the variant name corresponds to the color of the AGM name, with the total number of T/F variants per AGM listed to the right of the name. Viruses indicated by the red arrows represent the viral species found in the original inoculum used to infect AGMs. The set-point AGMs (AGM129-AGM132) represent viral diversity from blood draws at 12 dpi. All sequences were aligned using MUSCLE Alignment implemented in Geneious (https://www.geneious.com/) and then manually inspected and optimized. Phylogenetic trees were based on nucleotide sequences and constructed using the neighbor-joining method with Tamura-Nei distance model.

All AGMs were challenged with a single dose of plasma taken from an acutely infected AGM, which was diluted to 1 × 10^7^ viral copies (26). We utilized single genome amplification (SGA) and phylogenetic analyses to enumerate the T/F viral variants that established infection in viremic AGMs to ensure the AGMs were not overdosed. We detected between 3–10 T/F variants in each AGM, consistent with a moderate dose that was likely to infect all AGMs after a single challenge (Figure 4). Phylogenetic analysis showed that these T/F viruses were genetically distinct from each other, clustering randomly in relation to the different viral lineages found within the inoculum (Figure 4).

### vRNA was found in the lamina propria and lymphoid aggregates of the rectum and distal colon following inoculation.

**Figure 5:**
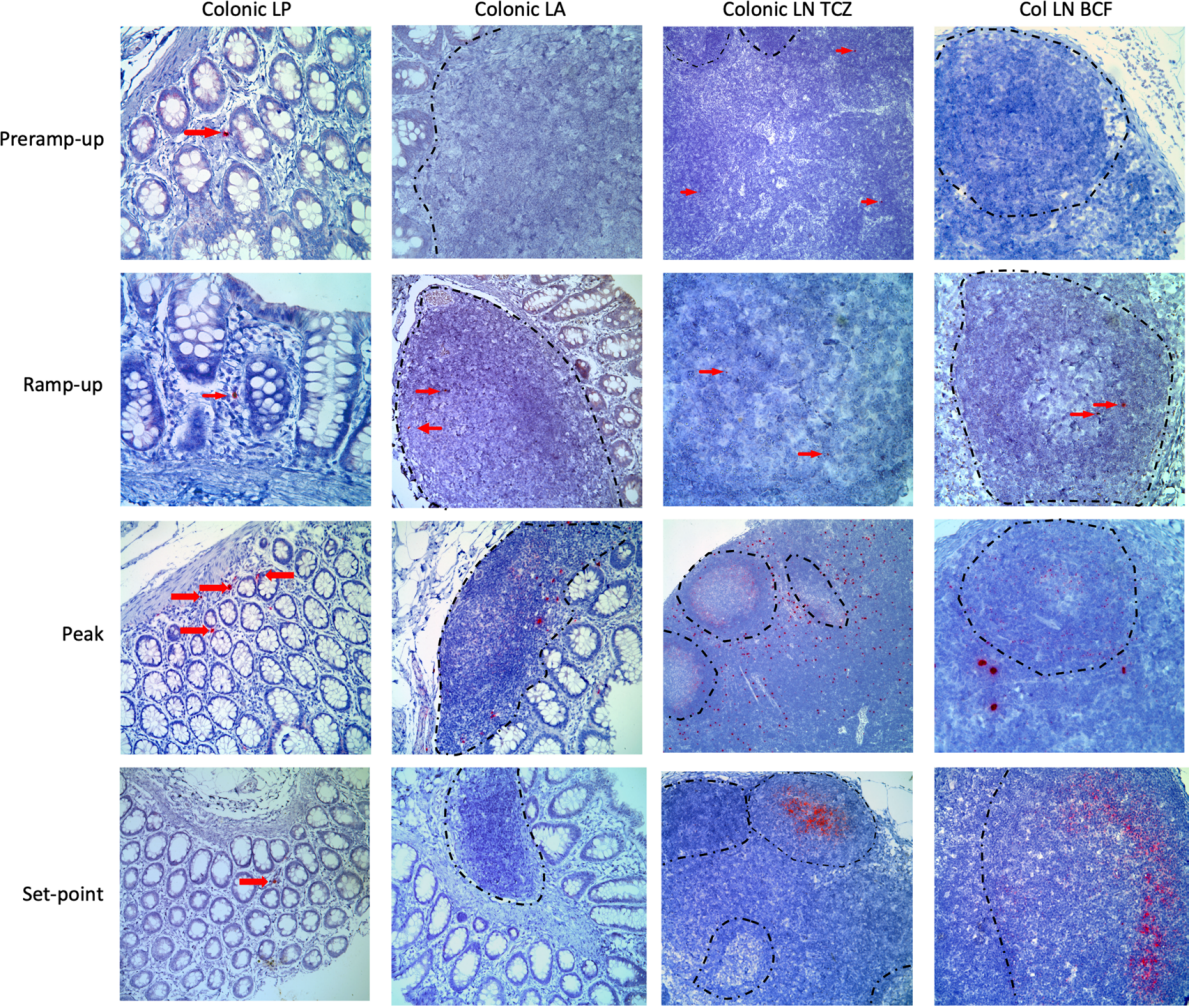
RNAScope for SIVsab RNA at the site of inoculation and in the draining lymphatics. RNAScope was performed on multiple sections of the rectum and the distal colon, which represent the site of inoculation. Only images from the distal colon are shown for consistency. Viral RNA was stained red, with the surrounding tissue counterstained purple. The red arrows point to foci of viral replication, especially at the earlier time points, when the virus is still rare. Each column represents a different tissue type, and each row represents a different time group. The BCFs in the LNs are all outlined with a dashed black line. All images were captured at 200× magnification with an Olympus FV10i confocal microscope. The time groups are shown on the left side of the figure, reflecting the status of viral replication, with: pre–ramp-up (1–3 dpi), ramp-up (4–6 dpi), peak (9–12 dpi), and set point (42 dpi). LP, lamina propria; LA, lymphoid aggregate; TCZ, T cell zone; BCF, B cell follicle.

We confirmed the qPCR detection of vRNA in the lamina propria of the rectum and distal colon and the colonic and distal LNs by RNAScope in situ hybridization (ISH) and histology (Figure 5). vRNA^+^ cells were detected in multiple pre–ramp-up AGMs, but these cells were rare and only in the lamina propria and the T cell zone of the colonic LNs. At this early stage of infection, these vRNA^+^ cells were typically single, isolated cells and not clustered foci of vRNA^+^ cells. During ramp-up, vRNA^+^ cells were found not only within the colonic lamina propria, but also in the colonic lymphoid aggregates in both the T cell zone and B cell follicle. Additionally, in these AGMs, vRNA^+^ cells were now found not only in the colonic LNs in the T cell zone and B cell follicles, but also in the more distal iliac LNs. The viral foci were still extremely rare, though, with the virus being detected less frequently in the lamina propria and lymphoid aggregates of the distal colon than in both the colonic and iliac LNs. In the samples tested, the virus was localized mostly to the T cell zone but also could be observed occasionally within the B cell follicles (Figure 5).

In AGMs necropsied at later time points of the acute infection, SIVsab was frequently found in both the lamina propria and lymphoid aggregates of the colon. We also observed large amounts of vRNA^+^ cells within the T cell zones of the LNs and abundant trapping of the virus by follicular DCs in the germinal centers (Figure 5). By the set point (>42 dpi), virus was detected rarely in the lamina propria and the lymphoid aggregates of the rectum and distal colon. In the LNs, large numbers of virions were trapped by follicular DCs, with little to no vRNA^+^ cells in the T cell zones (Figure 5). Overall, SIVsab detection by RNAScope supported both the virus-forming isolated foci of replication at the site of inoculation and the qPCR results showing early and rapid viral dissemination.

### SIVsab target cells at the site of inoculation are CD4^+^ T cells and myeloid cells.

**Figure 6:**
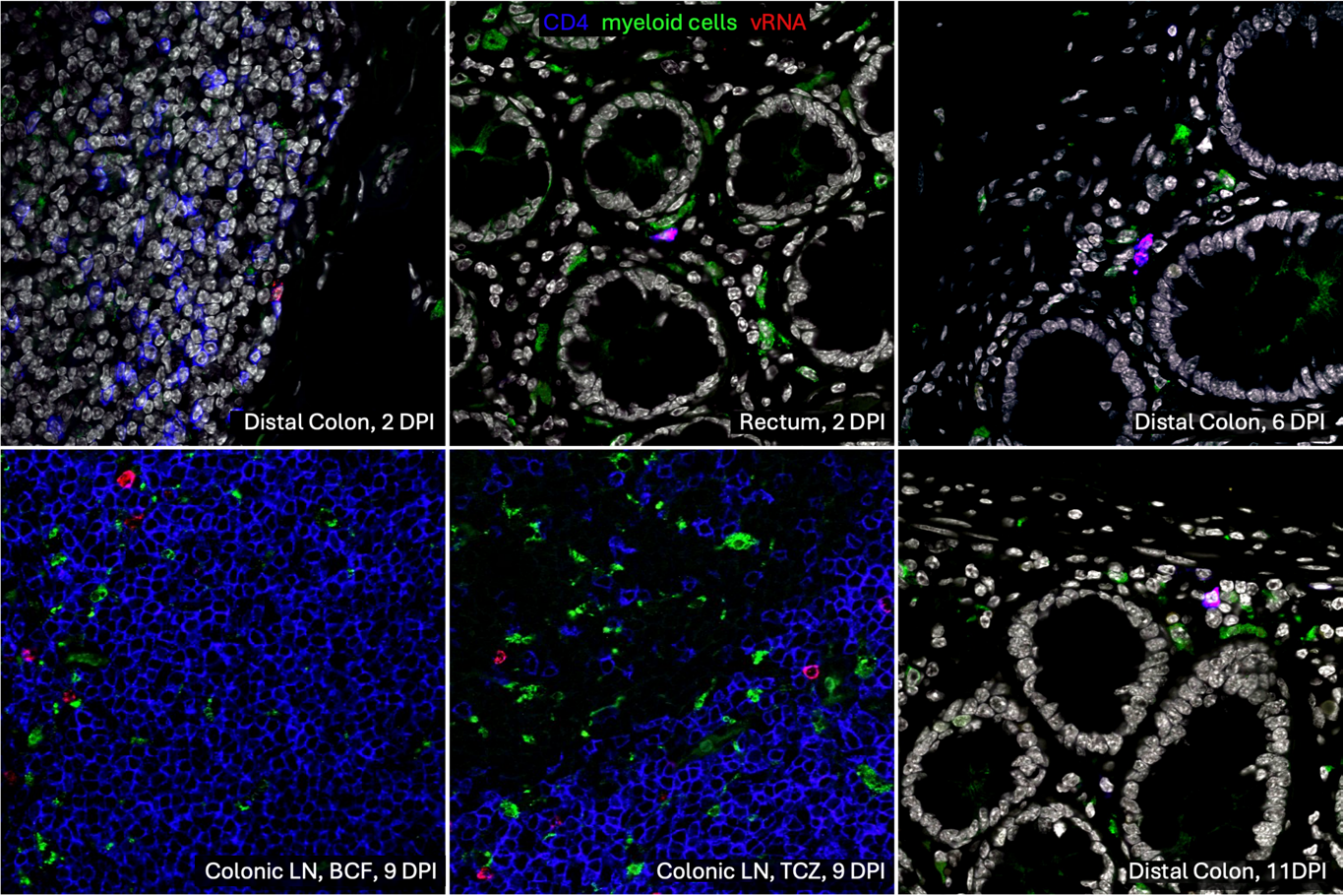
RNAScope for SIVsab RNA combined with immunofluorescence for CD4^+^ T cells and myeloid lineage cells. Combined RNAScope with immunofluorescence was used to identify with what cells the SIVsab RNA genomes colocalize. Viral RNA copies are shown in red, CD4^+^ T cells are shown in blue, and myeloid lineage cells (CD68^+^CD163^+^HAM56^+^) are shown in green. The tissue type and dpi are shown in white in the lower right corner of each image. All images were captured at 600× magnification with an Olympus FV10i confocal microscope using a 60× phase contrast oil-immersion objective and by imaging using sequential mode to separately capture the fluorescence from the different fluorochromes. For the colonic LN, both the B cell follicle (BCF) and T cell zone (TCZ) are shown.

The primary targets of both HIV and SIV are CCR5^+^CD4^+^ T cells (1, 22, 55–58). However, SIVsab is multitropic, as it can use multiple coreceptors, including CCR5, CXCR4, and the alternative coreceptor CXCR6 (7, 12, 59). To establish the identity of the target cell population that supported initial infection at the site of inoculation, rectum and distal colon sections were tested with a combined RNAScope and immunofluorescence assay specific for SIVsab vRNA, and they were costained for CD4^+^ T cells and myeloid lineage cells (CD68^+^ CD163^+^ HAM56^+^) in all AGMs (Figure 6). In all the tissues tested, vRNA^+^ cells always colocalized with CD4^+^ T cells but not with myeloid lineage cells. In the very early stages of infection (2 dpi), only rare single SIV-infected CD4^+^ T cells could be identified, though they were found to be present in both the rectum and the distal colon. Furthermore, the samples collected later in infection, when cells were infected through either direct local transmission or through systemic seeding, also showed that vRNA^+^ cells were consistently only CD4^+^ T cells. This strongly indicates that the T/F viruses are preferentially infecting CD4^+^ T cells resident to lamina propria of the rectum and distal colon, as was also reported for the HIV T/F strains (60–62).

### No significant alterations in the number of target cells at the site of inoculation.

**Figure 7:**
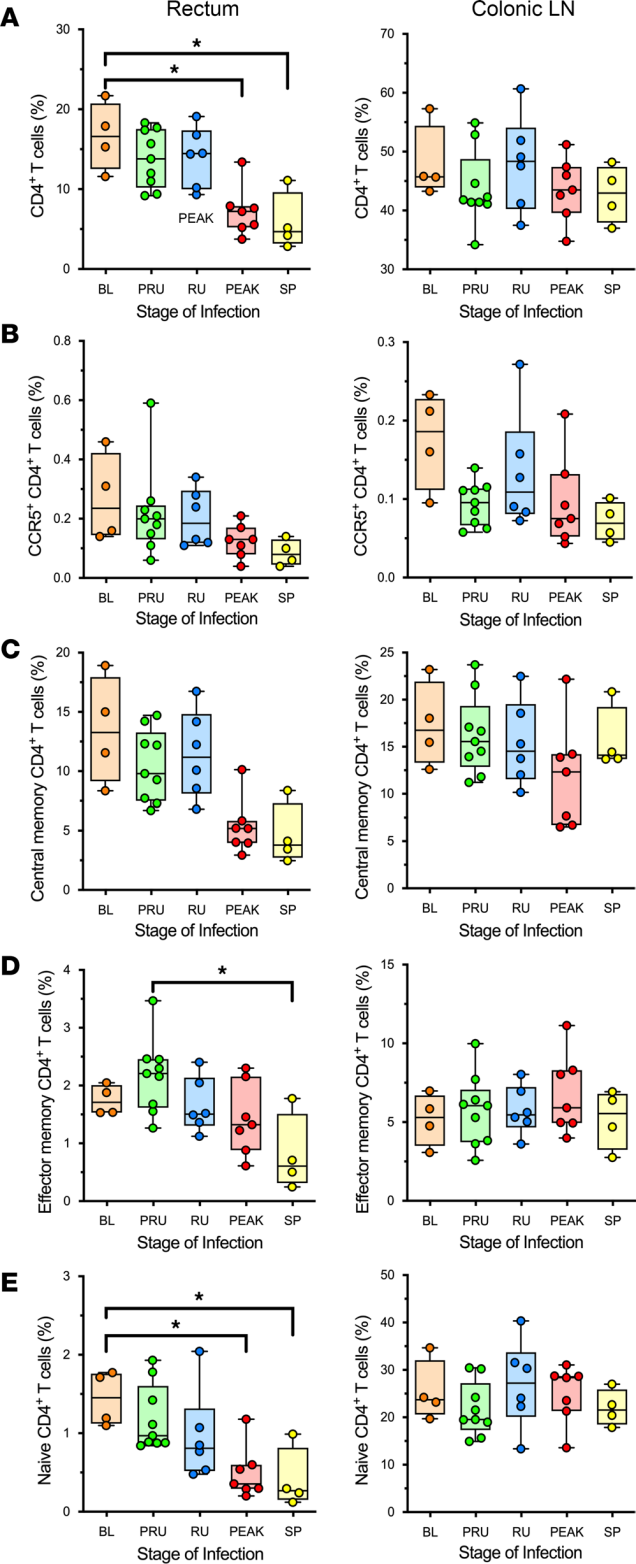
CD4^+^ T cell populations and subsets at the site of inoculation in SIVsab-infected African green monkeys (AGMs). (**A**–**E**) Percent populations of the total CD4^+^ T cells (**A**), CCR5^+^CD4^+^ T cells (**B**), CD4^+^ central memory (CD28^+^CD95^+^) (**C**), CD4^+^ effector memory (CD28^–^CD95^+^) (**D**), and CD4^+^ naive cells (CD28^+^CD95^–^) (**E**) in the rectum and colonic LN. The data are shown as box-and-whisker plots displaying the median, 1st and 3rd quartiles, and the minimum/maximum outliers, with individual points representing each AGM (*n* = 31). The 5 groups are based on dpi with the color codes: BL (baseline, preinfection, orange), PRU (pre–ramp-up, 1–3 dpi, green), RU (ramp-up, 4–6 dpi, blue), PEAK (peak, 9–12 dpi, red), and SP (set point, 46–55 dpi, yellow). An unpaired nonparametric Kruskal-Wallis test, followed by a Dunn’s multiple means comparison was used, with asterisks indicating statistical significance when compared with baseline values, with *P < 0.05. Brackets are used to indicate between which time groups there is a significant difference.

Having established that CD4^+^ T cells at the site of inoculation are the primary target cells of SIV, CD4^+^ T cells isolated from the rectum at the time of necropsy were analyzed by flow cytometry to assess the changes occurring in response to SIV infection. Overall, CD4^+^ T cells showed a significant decrease from the baseline levels by the peak of viral replication (*P* > 0.0397). By early chronic infection, the total CD4^+^ T cell population at the site of inoculation had yet to recover, as it remained significantly decreased compared with baseline levels (*P* > 0.0383) (Figure 7A). The levels of CCR5^+^CD4^+^ T cells (Figure 7B) and central memory CD4^+^ T cells (Figure 7C) in the rectum were not significantly lowered, but both showed a strong trend toward an overall reduction in population by early chronic infection. However, rectal effector memory CD4^+^ T cells were significantly depleted between the pre–ramp-up time points and the establishment of set point viral replication (*P >* 0.0253) (Figure 7D). Similarly, naive CD4^+^ T cell population was significantly decreased by the peak of viral replication (*P* > 0.0497) and into early chronic infection (*P* > 0.0493) (Figure 7E). By contrast, in the colonic LN, which is a draining LN of the site of inoculation, both the overall CD4^+^ T cell population (*P* > 0.99) and the subsets (effector memory: *P* > 0.99; central memory: *P* > 0.99; naive: *P* > 0.99) did not change significantly (Figure 7, A and C–E). The CCR5-expressing CD4^+^ T cells decreased by the peak of infection in the colonic LNs, as well, though without reaching significance (*P* > 0.1266) (Figure 7B).

### Immune cell alterations following infection.

The overall CD8^+^ T cell population and the central memory CD8^+^ T cells were relatively stable in both the rectum and the colonic LN throughout infection (Supplemental Figure 2, A and B). The effector memory CD8^+^ T cells increased significantly from the ramp-up to the peak (*P* > 0.0177) (Supplemental Figure 2C). In the colonic LNs, the naive CD8^+^ T cells were significantly altered between the pre–ramp-up to the peak (*P* > 0.0476) (Supplemental Figure 2D). Additionally, we saw no change in the population of CD8αα T cells in response to infection (Supplemental Figure 5). While these cells can provide similar functions to CD4^+^ T cells and can be upregulated in response to CD4^+^ depletion, our previous study of this subpopulation showed no significant change in overall cell counts or functionality of CD8αα T cells in response to experimental CD4^+^ depletion in the gut mucosa of uninfected AGMs (63).

By comparison, in the rectum, the levels of CD20^+^ B cells resident at the site of inoculation showed a declining trend over the course of infection, though this trend was not significant (*P* > 0.054) (Supplemental Figure 3A). There was also no significant change of the resident CD20^+^ B cell populations in the colonic LNs, though they did exhibit a tendency to increase by early chronic infection *(P* > 0.99), as observed in our previous studies. Furthermore, the innate immune effector myeloid DCs, plasmacytoid DCs, and macrophages were not significantly altered during acute infection in both the colonic LNs and the rectum (Supplemental Figure 3, B–D). The sole exception was represented by the NK cells, which significantly increased during the ramp-up of viral replication (*P* > 0.0093) (Supplemental Figure 3E). It should be noted that, due to a need to prioritize a limited number of cells for flow cytometry, some animals had no cells to stain for the DCs, macrophages, or NK cells, and this weakened our analysis for those populations.

## Discussion

While AGMs and other natural hosts of SIVs have evolved a number of adaptions to prevent disease progression to AIDS, they do not appear to be able to suppress viral replication during infection; furthermore, adult AGMs are unable to prevent transmission despite their limited availability of target cells, particularly at the mucosal sites (16, 22, 45, 45, 49, 64–67). By using AGMs as a good candidate model for mucosal HIV transmission that approximates all the stages of transmission while maintaining immune cell homeostasis upon SIV infection, our previous studies show that AGMs avoid progression to AIDS (48, 49, 26). By studying the earliest events of SIV infection in AGMs, we expected to gain insight on how natural hosts are permissive for SIV transmission and large-scale viral replication, without triggering immune reactions that ultimately lead to disease progression.

Our study has shown that, upon IR challenging AGMs with SIVsab, the virus spreads rapidly from the rectal site of inoculation to the blood via the lymphatic tissues. Accordingly, vRNA could be detected in plasma as early as 2–3 dpi, being consistently detected by 4 dpi in the majority of the monkeys. These results indicate that, upon IR inoculation, viral replication and dissemination occur nearly concurrently. This pattern is different from what was previously reported for the intravaginal transmission of SIVmac to RMs, where a delay in viral dissemination to the LNs from the site of inoculation was reported to create a “window of opportunity” for interventions aimed at blocking the virus spread (68, 69). Numerous approaches based on this “window of opportunity” were proposed to help block virus spread in humans, yet they were not particularly effective (31, 70). The very rapid and concomitant spread and amplification of the virus in the early infection is not specific to the natural hosts, as it was reported following IR inoculation of RMs (40, 71). The lack of a delay in virus spread (i.e., of opportunities for intervention) — together with previous studies (51, 52, 54, 72) showing that, during these very early stages of dissemination, SIV is able to gain access even to immune privileged sites, like the brain (Figure 2 and Figure 3) — indicates that any intervention has to block the virus at the site of entry as early as possible.

Our results also show that viral dissemination was not only rapid, but also pervasive. Virus was detectable in virtually every tissue surveyed by the ramp-up and peak stages of infection. In general, outside the site of inoculation, positivity of viral detection in tissues followed the initial detection of viremia, suggesting virus seeding via the bloodstream (most likely as both free virus and through cell trafficking). One complicating factor in the survey of the tissue VLs was the presence of blood within the tissue. The AGMs were not perfused with saline at the time of necropsy, and no special measures were taken (e.g., washing or rinsing) to remove the blood from the tissue sections prior to snap freezing. Unknown volumes of blood, containing both cell-associated virus and free virus, could have contributed to the total number of viral copies measured by the qPCR. However, it is unlikely that blood contamination drastically altered the tissues quantification results because some highly vascularized tissues, such as heart, spleen, and stomach, exhibited high VLs while other highly vascularized tissues, such as kidney, liver, and lung, had relatively low or average vRNA loads.

The main objective of this study was to focus on the earliest viral dynamics at the rectal site of inoculation. Previous studies reported that immediately following vaginal transmission, after crossing the mucosal epithelium, the virus infects resident target cells and forms small foci where it is amplified prior to its systemic dissemination (31, 35, 37). To identify the initial foci of viral replication after IR transmission, we excised the rectum and distal colon together; we then sectioned the 2 tissue segments into strips and divided these further into pieces. Each piece was used for a different assay, as assigned on a rotating basis, such that no 2 contiguous pieces would be used for the same assay. The purpose of this sampling strategy was to capture any of these initial foci of viral replication, where the virus has infected only a handful of target cells. This approach was successful, as multiple pieces of the rectum and distal colon were positive for SIVsab RNA and DNA within 1–3 dpi following inoculation. This very early detection of virus at the site of inoculation suggests that, in the natural hosts, the population of founder cells is quickly established following inoculation, as was also reported in macaques (40, 73–75).

In addition to viral replication at the site of virus exposure, SIVsab RNA was also detectable in the draining colonic LNs as early as 2–3 dpi. Early detection of the virus in the draining LNs of the portal of entry is not surprising and supports the rapid spread of SIVsab through the lymphatics and into circulation. In the LNs, virus initially occupied only the T cell zone, with little to no trapping by follicular DCs occurring until after the ramp-up period, which enabled the virus to circulate unimpeded through the lymphatic, and gain rapid access to the bloodstream via the thoracic duct (76). Another possible explanation for the rapid virus dissemination following IR inoculation (much faster than what was previously reported for intravaginal transmission in macaques and in line with IR transmission in RMs; refs. 68, 69, 75) is that the rectum, with its thin, single-layered epithelium, provides a more permissive route of virus entry, particularly if there are preexisting microabrasions or breakages (31, 40). Additionally, the lamina propria of the rectum and distal colon are densely populated with lymphoid aggregates containing many activated CD4^+^ T cells, and this could facilitate early viral replication as well as provide more immediate access to the lymphatic system. However, our inoculation strategy was atraumatic, and no physical damage to the gut epithelium was observed in any of our histological surveys (data not shown).

The higher frequency of target cells in the rectum and colon compared with vaginal mucosa may explain why our T/F virus analysis found that, while a mucosal bottleneck occurred, it was not as stringent as during natural transmission in AGMs, being more similar to experimental IR transmission in RMs (26, 45, 68, 69). Indeed, we identified 3–10 T/F strains in each AGMs, which is more than the 1–3 strains transmitted in both humans (43, 44) and previous AGM studies (26, 45, 77) upon IR exposure. However, the most likely explanation for these discrepancies is a slight overdosing in this study to maximize the odds of infection. Such a strategy was necessary due to a serial necropsy schedule where infection status would be unknown until postmortem. By slightly increasing the infectious dose to allow an effective transmission upon single exposure, we attempted to avoid euthanizing uninfected AGMs during the very early stages of infection. In this respect, we largely succeeded, since out of the original 29 AGMs that were challenged with SIVsab, only 2 did not become infected and had to be excluded from the study, highlighting the importance of optimizing the dosage to increase the likelihood of infection.

Finally, to complete the characterization of SIVsab IR transmission, the target cells of the virus immediately following transmission were determined using RNAScope combined with immunofluorescence. vRNA^+^ cells were shown to be CD4^+^ T cells, but not myeloid lineage cells (CD68^+^CD163^+^HAM56^+^), in agreement with previous studies that reported that T/F HIV strains exclusively target T cells and not macrophages (60–62). This was expected, as our stock was generated from diluted plasma taken from an acutely infected AGM. Thus, it contained largely T/F viruses, which were reported to exclusively infect lymphoid and not myeloid cells, unlike the viral lineages that emerge later during infection (78, 79).

Overall, the characterization of the early events of SIVsab replication and dissemination following transmission showed that, during IR transmission, the virus is systemically spread rapidly from the site of inoculation, possibly moving unabated through the draining lymphatics into the bloodstream, where it is disseminated throughout the body. In this sense, we found very little difference between the patterns of viral replication and spread between progressive and natural hosts following IR inoculation. This suggests that the speed of the viral spread has little effect on the development of disease progression, at least in the case of IR transmission.

It is worth noting that the rapidity with which SIVsab disseminates systemically after IR transmission is of additional importance to HIV research, as it has been previously suggested that a “window of opportunity” exists during the eclipse phase of viral replication in HIV that represents the best chance to target the virus with a vaccine, microbicide, or some other intervention to prevent infection. This window was thought to occur only prior to establishment of systemic infection (31, 70), though rapid establishment of viremia following IR transmission had already been demonstrated in RMs (68, 69). If SIVsab replication and dissemination in AGMs are indeed more similar than dissimilar to what has been observed in HIV and SIV infection of RMs following IR exposure, this window of opportunity to prevent systemic infection may by extremely short or even nonexistent, even in a natural host. As such, our results suggest that measures to completely block transmission of the virus at the site of entry will be more useful than trying to curtail the infection after it is established via the rectal route.

## Methods

### Sex as a biological variable.

For this study, only male AGMs and the IR route of infection were used to avoid the physiological variability of the vaginal mucosa associated with the estrus cycle (80), even though vaginal transmission is far more prevalent in wild NHPs. We do not anticipate these findings to be directly applicable to the other sex, unless a similar IR route of transmission is employed.

### Animals and infections.

Thirty-three adult (4–9 years old) male AGMs (*Chlorocebus sabaeus*) of Caribbean origin were used in this study. Of the initial 33 AGMs, 4 AGMs served as uninfected controls, while the remaining 29 AGMs were IR challenged with infectious plasma originally collected from an acutely infected AGM, diluted to contain 1 × 10^7^ RNA copies of SIVsab (Supplemental Figure 1) (48). This dosage was established during a preliminary study as a moderate to low dose that could reliably infect adult AGMs IR (26). Infectious acute plasma was chosen as an inoculum to better reflect the natural viral diversity in the wild; it is also more infectious than plasma taken from chronically infected NHPs (81). Given the nature of the study, it was necessary to perform serial necropsies of AGMs to obtain the tissues required for proper characterization of the site of inoculation, the virus spread throughout the body and a wide variety of different immune responses. Longitudinal biopsies would have provided neither the quantity nor the diversity of tissues necessary for such an extensive survey.

### Tissue sampling and isolation of mononuclear cells.

At the necropsy, we collected the maximum amount of blood from each animal. Extensive tissue sampling also was performed. Sections of numerous tissue sites were collected for snap freezing in liquid nitrogen for DNA/RNA extraction or for fixation in 4% paraformaldehyde for ISH/FISH. Additional tissue samples were collected for lymphocyte separation from specific tissues of interest, including rectum, colon, ileum, jejunum, and a variety of regional LNs (colonic, iliac, obturator, mesenteric, axillary, inguinal, and submandibular). Cells were separated from whole blood and tissues and were used fresh for flow cytometry. Any remaining cells were frozen for later use at –80°C in freezing medium containing heat-inactivated FBS with 10% dimethyl sulfoxide (DMSO).

Special attention was paid to collection of the tissue from the site of inoculation: the rectum and the distal colon. These tissues were removed first, prior to any other tissue compartment; opened longitudinally; and then sectioned into 6 strips each, for a total of 12 strips. These strips were then divided into 5 approximately 1 cm^2^ pieces. The pieces were then collected for various purposes, including IHC, RNAScope, immunofluorescence, and DNA/RNA extraction for PCR. Since the initial foci of viral replication within the site of inoculation can be widely distributed, the pieces were taken using a rotating collection strategy to maximize the probability of capturing 1 or more of these foci.

Plasma was separated from whole blood within 1 hour of collection, as described (82). Peripheral blood mononuclear cells (PBMCs) were then isolated, as described (48, 83). Cells were separated from LNs, as described (84, 85). Cells were also isolated from the gut sections taken during the necropsy, which were processed as previously described (26, 58).

### Plasma vRNA extraction and cDNA synthesis.

vRNA was extracted from plasma using a QIAGEN viral RNA Mini kit (QIAGEN). RNA was then eluted, and reverse transcription was preformed using a TaqMan Gold Reverse Transcription PCR (RT-PCR) kit and random hexamers (Applied Biosystems), as described (48). The RT-PCRs were run in a Gene Amp PCR System 9700 thermocycler (Applied Biosystems).

### Plasma and tissue VL quantification and SCA.

Plasma and tissue VLs were monitored by qPCR based on a 180 bp segment located in the *gag* region, as described (17, 25, 49), using primers and probes specifically designed for SIVsab (48, 49), which were synthesized by Integrated DNA Technologies (IDT). All qPCRs were run in duplicate, and negative controls for the RT-PCR and qPCR were included on each plate. The qPCRs were run on 7900HT Fast Real Time System (Applied Biosystems), as described (49). qPCR was performed by using TaqMan Gene Expression Master Mix (Applied Biosystems), as described (82, 86), using previously published SIVsab-specific primers and probes (16, 24). Absolute vRNA copy numbers were calculated relative to amplification of an SIVsab standard, which was subjected to RT-PCR in parallel with the samples being tested. The standard was generated as described (49). The detection limit of this conventional qPCR was 30 vRNA copies/mL of plasma (49).

To detect the virus as early as possible, we employed SCA for quantification of SIVsab (82, 86). Large volumes of plasma samples (7.5 mL) were used in SCA (82, 87). All samples were run in triplicate on 96-well plates. Any of the samples that initially tested negative were retested, as the levels of circulating virus during very early acute infection (primarily during the pre–ramp-up [1–3 dpi]) were anticipated to be low (50, 86). For the same reason, when large volumes of plasma were available from AGMs euthanized at 1–3 dpi, SCA was performed multiple times.

### CSF VL quantification.

CSF RNA extraction and PCR were performed with the same methodology described above for plasma but using slightly different reagents. vRNA was extracted from CSF with the QIAGEN viral RNA Mini kit, followed by reverse transcription. Instead of the Taqman Gold Reverse Transcription kit, the M-MLV reverse transcriptase (Thermo Fisher Scientific) was used with random primers (Promega) and RNasin ribonuclease inhibitor (Promega), as per the manufacturer’s specifications.

### SGA of T/F viruses.

SGA was performed as described (26, 43, 45). Both DNA strands were sequenced, and chromatograms were inspected at every position for double peaks, which would be indicative of a Taq polymerase error in an early cycle or PCR priming from more than 1 template. Sequences with mixed bases were excluded from further analysis as previously described (88, 89).

### Tissue DNA/RNA extraction.

DNA/RNA was extracted from tissue sections snap frozen in liquid nitrogen. Prior to thawing, the mass of each tissue section was measured and was used to estimate the necessary volume of TriReagent (Molecular Research Center) at a ratio of 100 mg/1 mL. The snap-frozen sections were transferred on dry ice from standard cryotubes to SPEX SamplePrep polycarbonate cryotubes (SPEX SamplePrep). Then, stainless steel ball bearings and hex nuts (MSC) were layered over each tissue before the addition of the required volume of TriReagent. The tissues were homogenized using a SPEX 2010 Geno/Grinder (SPEX SamplePrep), with a single cycle of agitation at 1,600 rpm for 2 minutes. Tissues that are more difficult to break down, such as heart and stomach, received 2 cycles of agitation. Following homogenization, the tissues were allowed to sit for 15–20 minutes in TriReagent to solubilize small tissue particles. Next, 1 mL of tissue lysate was transferred from each sample to 1.5 mL snap cap tube. The RNA was extracted first by adding 100 μL bromochloropropane (BCP) (MRC) to each sample, vortexing for 30 seconds, and then spinning at 14,000*g* for 15 minutes at 4°C. After centrifugation, the upper aqueous phase was removed and transferred to a tube with 12 μL of 20 mg/mL glycogen (Sigma-Aldrich) before being mixed with 500 μL isopropanol. The solution was then spun at 21,000*g* for 10 minutes at room temperature (RT), the supernatant was removed, and the pellet was washed with 600–800 μL 70% ethanol. Each pellet was allowed to sit under ethanol for 3 days at –20°C before being dried and either immediately resuspended or stored at –80°C for future use. The same protocol was repeated for DNA, except with 500 μL DNA Back Extraction solution (4 M GuSCN, 1M Tris base, 50 mM sodium citrate) instead of BCP.

### RNAScope ISH.

To visualize early virus replication, RNAScope was performed to detect SIVsab mRNA transcripts in formalin-fixed paraffin-embedded (FFPE) tissues using probes specific for SIVsab (Advanced Cell Diagnostics) and the 2.0 HD RED reagent kit (Advanced Cell Diagnostics) following the manufacturer’s instructions with modifications, as previously described (90, 91). Briefly, FFPE sections were baked and deparaffinized. Tissue-specific pretreatment was performed according to the manufacturer’s instructions for each tissue type, followed by multiple hybridization steps at 40°C. The slides were finally counterstained using CAT Hematoxylin (Biocare Medical) to visualize nuclei. The slides were then imaged at 200× magnification with an Aperio AT2 system (Leica Biosystems).

### RNAScope combined with immunofluorescence.

To establish the identity of the SIVsab target cells, RNAScope with immunofluorescence was performed as previously described (92, 93). Briefly, we combined the RNAScope assay with immunofluorescence staining targeting CD4^+^ T cells using anti-CD4 antibody (rabbit; 1:100, Abcam) and targeting myeloid lineage with a combination of anti-CD68 antibody (mouse; 1:500, Novocastra), anti-CD163 antibody (mouse; 1:500, Biocare), and anti-HAM56 antibody (mouse; 1:1,000, Dako) (Supplemental Table 1). Slides were rinsed in TBS-Tween-20, incubated with donkey anti–mouse Alexa Fluor 488, and donkey anti–rabbit Alexa Fluor 647 (Invitrogen) for 1 hour at RT in the dark and rinsed with TBS-Tween-20. Slides were incubated with 0.1% Sudan Black B in 70% ethanol (ENG Scientific) and 1× TBS for 30 minutes at RT to quench autofluorescence; they were then incubated with 300 nM DAPI for 10 minutes. Slides were rinsed, mounted with Prolong Gold (Invitrogen), and imaged on an Olympus FV10i confocal microscope (Olympus) (92). All images were captured at 600× magnification using a 60× phase contrast oil-immersion objective and by imaging using sequential mode to separately capture the fluorescence from the different fluorochromes.

### Flow cytometry.

Cells were stained as described (48, 85). The absolute counts of peripheral blood lymphocytes were determined by using TruCount tubes (BD Bioscience), as described (82, 94). Blood CD45^+^ cells were quantified using 50 μL whole blood stained with antibodies in TruCount tubes that contained a predefined number of fluorescent beads to provide internal calibration. The CD4^+^ and CD8^+^ T cell counts were then calculated using the ratio of CD4^+^ and CD8^+^ T cells to CD3^+^ cells in the whole blood at the same time point.

Immunophenotyping of the immune cells isolated from blood, LNs, and intestine was performed using fluorescently conjugated mAbs (Supplemental Table 2), chosen to characterize a wide range of immune cell types and markers. For the adaptive cell type panels, dead cells were omitted by gating first for singlets. An example of a gating strategy for primary T cell populations (CD3^+^, CD4^+^, CD8^+^) and T cell subsets (CCR5^+^, CD8αα^+^, effector memory, central memory, naive) is shown in Supplemental Figure 4, with data from colonic LN cells isolated from one of the uninfected AGMs. Data were acquired on an LSR-II flow cytometer (Becton Dickinson) and analyzed using the Flowjo software version 10.10.0 (Tree Star Inc.).

### Statistics.

To compare changes in the immune cell populations between preinfection and postinfection time points from the same AGMs, we used the nonparametric 2-tailed Wilcoxon matched-pairs signed-ranks test, with a *P* ≤ 0.05. For comparisons between the flow cytometry results from the control and infected AGMs, we used the unpaired nonparametric Kruskal-Wallis test, followed by a Dunn’s multiple means comparison test to correct for multiple comparisons. The family-wise significance and confidence levels were set at 0.05. All tests were performed using the Graphpad Prism 10 Software (Graphpad Software Inc.).

### Study approval.

All AGMs were housed at the RIDC animal facility of the University of Pittsburgh, an AAALAC International facility, as per the regulations outlined in the *Guide for the Care and Use of Laboratory Animals* (National Academies Press, 2011) (95) and the Animal Welfare Act (96). All animal experiments were approved by University of Pittsburgh IACUC (protocol no. 1008829) Efforts were made to minimize NHP suffering, in agreement with the recommendations in *The Use of Nonhuman Primates in Research* (97). The NHP facility was air conditioned, with an ambient temperature of 21°C–25°C, a relative humidity of 40%–60%, and a 12-hour light/dark cycle. AGMs were socially housed in suspended stainless-steel wire-bottomed cages. A variety of environmental enrichment strategies were employed, including providing toys to manipulate and playing entertainment videos in the NHP rooms. The NHPs were observed twice daily, and any signs of disease or discomfort were reported to the veterinary staff for evaluation. At the end of the study, the NHPs were euthanized following procedures approved in the IACUC protocol.

### Data availability.

All data used in the paper are available in the Supporting Data Values file.

## Author contributions

Study design, management of the animal procedures, and protocol oversight were done by KDR, CA, and IP. Manuscript preparation was done by KDR, JDE, BFK, CA, and IP. Standard plasma viral load optimization and analysis were done by DM. SCA implementation, testing, and analysis were done by BBP. DNA/RNA extractions from whole tissues were done by KDR and DP. All tissue viral load testing and analysis was done by KDR. Staining of immune cell populations for flow cytometry was done by CX, KW, and EBC. Flow cytometry data analysis was done by KDR and CX. Tissue RNAScope and immunofluorescence were performed by CD. Histological preparation of tissues and slides used for RNAScope and immunofluorescence were done by CD. SGA was performed by BFK.

## Supplementary Material

Supplemental data

Supporting data values

## Version 1 12/06/2024

Electronic publication
