# Supplementary material for: Rapid systemic spread and minimal immune responses following SIVsab intrarectal transmission in African green monkeys
Source: JCI Insight. 2024 Dec 6;9(23):e183751. doi: 10.1172/jci.insight.183751 (PMC11623940; doi:10.1172/jci.insight.183751)
Supplement: Supplemental data [file jciinsight-9-183751-s150.pdf]

Rapid Systemic Spread and Minimal Immune Responses Following SIVsab Intrarectal Transmission in African Green Monkeys

----

Kevin D. Raehtz,[1] Cuiling Xu,[1,2] Claire Deleage,[3] Dongzhu Ma,[2] Benjamin B. Policicchio,[4] Egidio Brocca-Cofano,[2#a] Daniele Piccolo,[5] Kathryn Martin,[1] Brandon F. Keele,[3] Jacob D. Estes,[3,#b,] Cristian Apetrei[2,4¶*] and Ivona Pandrea,[1,4¶*]

SUPPLEMENTAL INFORMATION

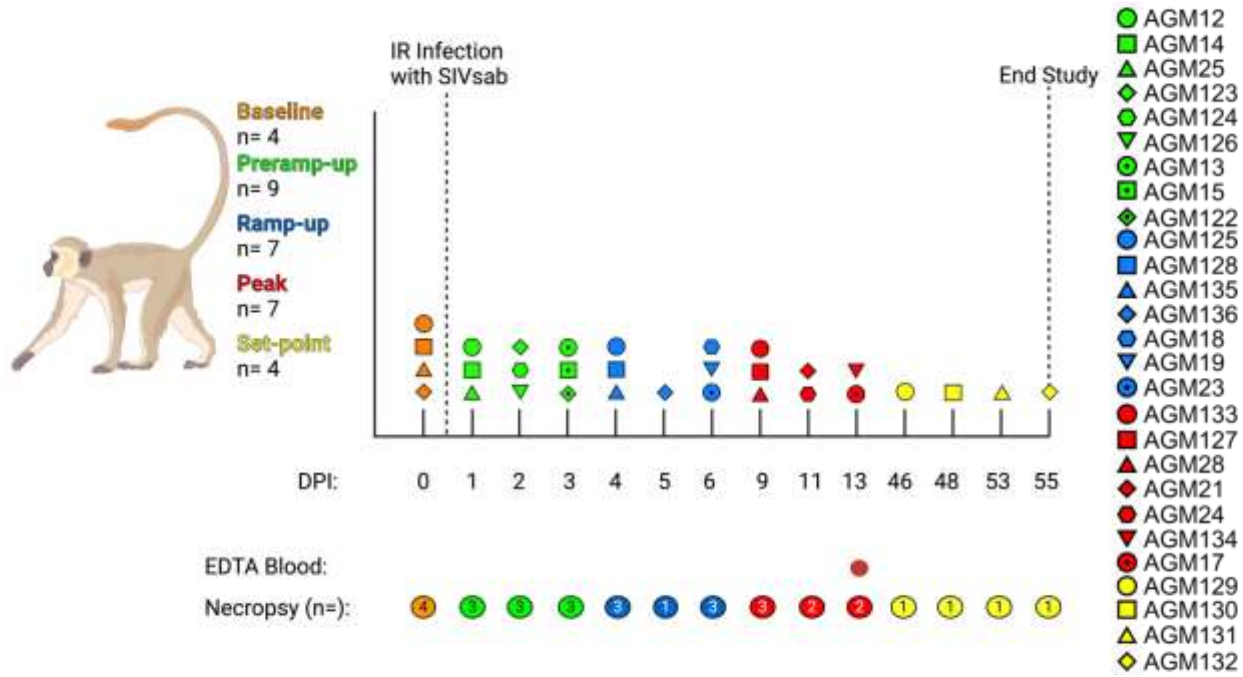

Supplemental Figure S1: Animal groups for study of the early events of intrarectal (IR) SIVsab infection. Thirty-one African green monkeys (AGMs) were included in this study (excluding the two AGMs which were inoculated, but uninfected). Twenty-seven AGMs were exposed IR to SIVsab92018 and became infected, while 4 AGMs were unchallenged as a control group. The AGMs were euthanized throughout the acute and early chronic SIV infection and were divided into the five groups based on their predicted viremic status at the day postinfection (DPI) that the necropsy was performed: (i) preinfection (orange, baseline); (ii) preramp-up (green, 1-3 DPI); (iii) ramp-up (blue, 4-6 DPI); (iv) peak (red, 9-13 DPI); (v) set-point (yellow, 46-55 DPI). Each AGM is assigned an individual symbol and color indicating at which stage of infection they were necropsied. The set-point animals also had EDTA blood drawn at the peak of infection, as well as at the time of their necropsies. Base Figure created with BioRender.com

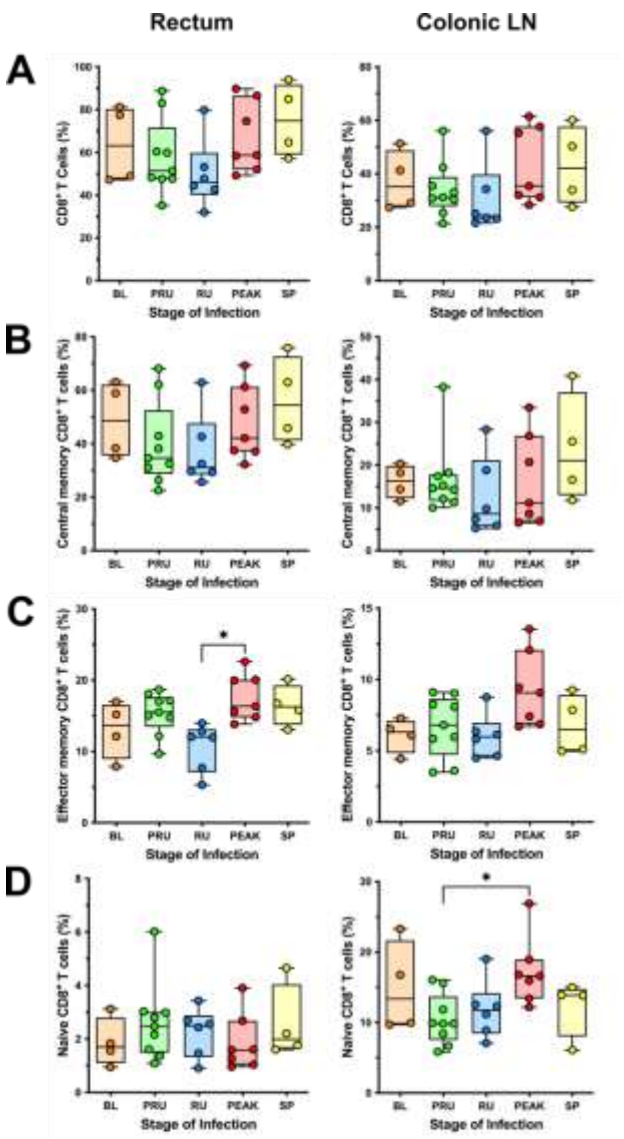

Supplemental Figure S2: CD8$^+$ T-cell populations and subsets at the site of inoculation in SIVsab-infected African green monkeys (AGMs). Percent populations of **(A)** the CD8$^+$ T cells, **(B)** CD8$^+$ central memory (CD28$^+$ CD95$^+$), **(C)** CD8$^+$ effector memory (CD28^{neg} CD95$^+$), and **(D)** CD8$^+$ naïve cells (CD28$^+$ CD95^{neg}) in the rectum and colonic LN. The data are shown as box-whisker plots displaying the median, 1st and 3rd quartiles and the min/max outliers, with individual points representing each AGM (n=31). The five groups are based on DPI with the color codes: BL (baseline, preinfection, orange), PRU (preramp-up, 1-3 DPI, green), RU (ramp-up, 4-6 DPI, blue), PEAK (peak, 9-12 DPI, red) and SP (set-point, 46-55 DPI, yellow). An unpaired nonparametric Kruskal-Wallis test, followed by a Dunn's multiple means comparison was used, with asterisks indicating statistical significance when compared to baseline values, with *=p<0.05. Brackets are used to indicate between which time groups there is a significant difference.

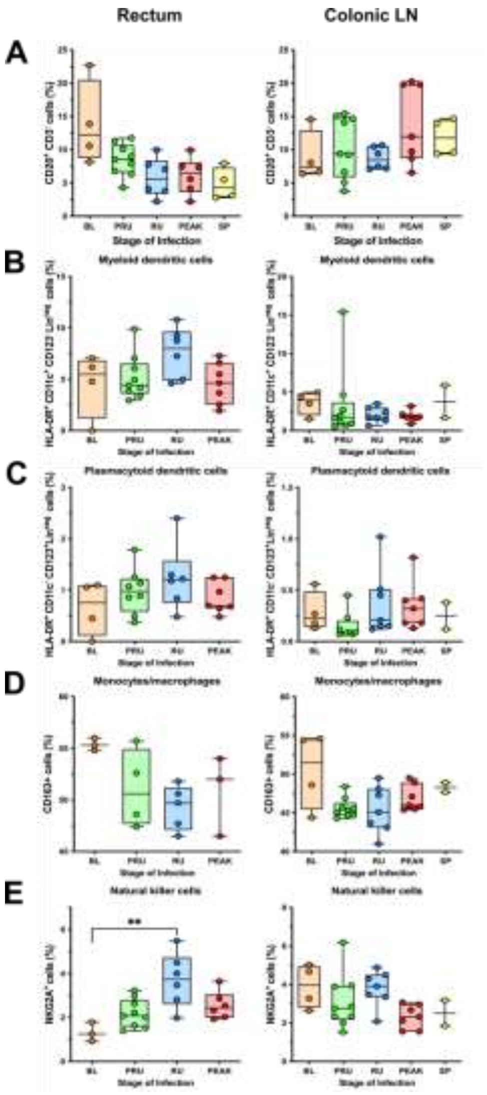

Supplemental Figure S3: CD20$^+$ B cells and innate immune cell populations at the site of inoculation in SIVsab-infected African green monkeys (AGMs). Percent populations of **(A)** the CD20$^+$ B cells, **(B)** myeloid dendritic cells (HLA-DR$^+$ CD11c$^+$ CD123^{neg} Lineage^{neg} [CD3^{neg} CD20^{neg} CD14^{neg}]), **(C)** plasmacytoid dendritic cells (HLA-DR$^+$ CD11c^{neg} CD123$^+$ Lineage^{neg} [CD3^{neg} CD20^{neg} CD14^{neg}]), **(D)** CD163$^+$ monocytes/macrophages and **(E)** NKG2A$^+$ natural killer cells in the rectum and colonic LN. The data are shown as box-whisker plots displaying the median, 1st and 3rd quartiles and the min/max outliers, with individual points representing each AGM (*n*=31); some data points are not included due to lack of cells in the original assay The five groups are based on DPI with the color codes: BL (baseline, preinfection, orange), PRU (preramp-up, 1-3 DPI, green), RU (ramp-up, 4-6 DPI, blue), PEAK (peak, 9-12 DPI, red) and SP (set-point, 46-55 DPI, yellow). An unpaired nonparametric Kruskal-Wallis test, followed by a Dunn's multiple means comparison was used, with asterisks indicating statistical significance when compared to baseline values, with *=p<0.05. Brackets are used to indicate between which time groups there is a significant difference.

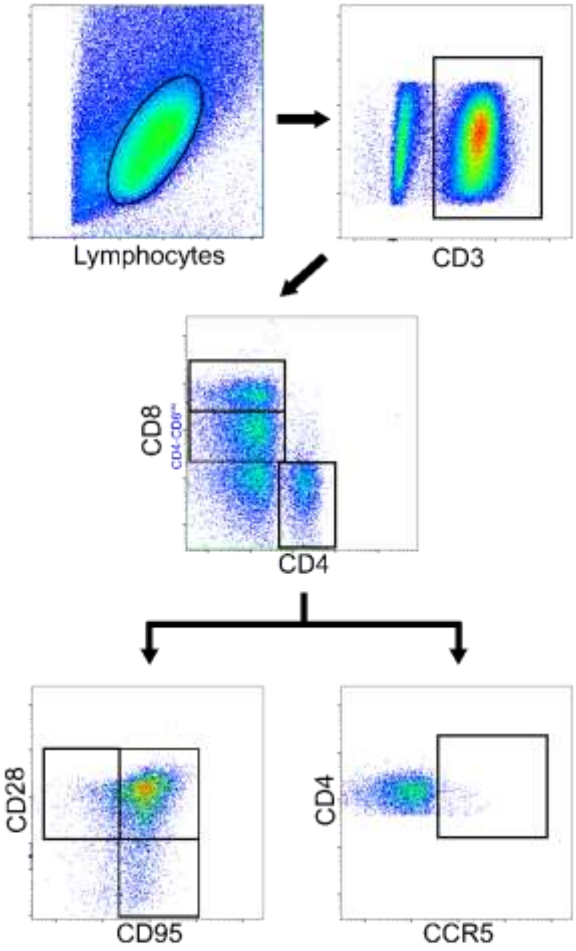

Supplemental Figure S4: Flow cytometry gating strategy for CD4$^+$ and CD8$^+$ populations. Gating strategy used to delineate primary T-cell populations (CD3$^+$, CD4$^+$, CD8$^+$) and T-cell subsets (CCR5$^+$, EM [CD95^{neg} CD28$^+$], CM [CD95$^+$ CD28$^+$], and naïve [CD95^{neg} CD28^{neg}]). Also shown is the gating for the CD8αα subpopulation within the total CD8$^+$ population. The example data shown is from cells isolated from the rectum of one of the uninfected AGMs. All gates were generated using Flowjo software version 10.10.0 (Tree Star Inc, Ashland, OR).

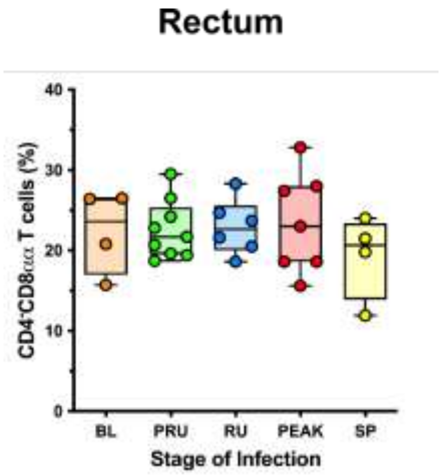

Supplemental Figure S5: CD8αα$^+$ T-cell populations and subsets at the site of inoculation in SIVsab-infected African green monkeys (AGMs). Percent populations of CD8αα T cells. The data are shown as box-whisker plots displaying the median, 1st and 3rd quartiles and the min/max outliers, with individual points representing each AGM (*n*=31). The five groups are based on DPI with the color codes: BL (baseline, preinfection, orange), PRU (preramp-up, 1-3 DPI, green), RU (ramp-up, 4-6 DPI, blue), PEAK (peak, 9-12 DPI, red) and SP (set-point, 46-55 DPI, yellow). An unpaired nonparametric Kruskal-Wallis test, followed by a Dunn's multiple means comparison was used, with asterisks indicating statistical significance when compared to baseline values, with *=$p<0.05$. Brackets are used to indicate between which time groups there is a significant difference.

Supplemental Table S1: RNAscope/IF Antibodies

Target Marker	Antibody	Clone	Reference #	Manufacturer	**Dilution**
CD68	Mouse IgG	KP1	GA60961-2	Agilent Dako	1:500
CD163	Mouse IgG	10D6	NCL-L-CD163	Novocastra	1:500
CD4	Rabbit IgG	EPR6855	ab133616	Abcam Inc.	1:100
HAM56	Mouse	HAM56	NA	Agilent Dako	1:1000

Table S2: Flow cytometry antibodies and fluorochromes

Antibody	Clone	Reference #	Manufacturer
CD3e-FITC	SP34	556611	BD Pharm
CD95-FITC	DX2	555673	BD Pharm
CD14-PE	M5E2	555398	BD Pharm
CD163-PE	GHI/61	556018	BD Pharm
CD95-PE	DX2	555674	BD Pharm
CCR5-PE (CD195-PE)	3A9	556042	BD Pharm
CD123-PE-Cy7	7G3	560826	BD Pharm
CD8 α/b -PE Texas Red	3B5	MHCD0817	Invitrogen
CD28-PE-Cy7	CD28.2	560684	BD Pharm
HLA-DR-PE-Cy7	L243	335813	BD BioSci
CD20-APC-Cy7	L27	335794	BD BioSci
CD20-APC-H7	2H7	560853	BD Pharm
CD4-APC	L200	551980	BD Pharm
NKG2A-APC	Z199.1	A60797	Beckman Coulter
HLA-DR-APC-Cy7	L243	335796	BD BioSci
CD3-V450	SP34-2	560351	BD Pharm
CD11c-APC	S-HCL-3	340544	BD BioSci
